# *Lentinus edodes* promotes fat removal in hypercholesterolemic mice

**DOI:** 10.3892/etm.2013.1333

**Published:** 2013-10-08

**Authors:** HYUN YANG, INHO HWANG, SUN KIM, EUI-JU HONG, EUI-BAE JEUNG

**Affiliations:** Laboratory of Veterinary Biochemistry and Molecular Biology, College of Veterinary Medicine, Chungbuk National University, Cheongju, Chungbuk 361-763, Republic of Korea

**Keywords:** *Lentinus edodes*, hypercholesterolemia, eritadenine, CYP7A1

## Abstract

*Lentinus (L.) edodes* (shiitake mushroom) is used as a traditional medicine in Asia. One of the components of *L. edodes*, eritadenine (an adenosine analog alkaloid), has been shown to reduce cholesterol levels. The hypocholesterolemic action of eritadenine appears to be achieved through the modification of hepatic phospholipid metabolism. In the present study, the effects of *L. edodes* in a mouse model of hypercholesterolemia were investigated. Hypercholesterolemia was induced by the consumption of a high-fat diet (HFD). The animals were divided into six groups, which were fed a normal diet, HFD alone, HFD containing eritadenine [10 mg/kg of body weight (BW)] or HFD with 5, 10 or 20% *L. edodes*, respectively, for 4 weeks (from 5 to 9 weeks of age). The mice in the six groups had similar BW gains. Total serum cholesterol (T-CHO), low-density lipoprotein (LDL) and triglyceride (TG) levels were increased in the HFD-fed group compared with those in the normal diet group. However, the levels of high-density lipoprotein (HDL) were not significantly altered. In mice treated with *L. edodes* (5, 10 or 20%), the T-CHO, LDL and TG serum levels were reduced in a dose-dependent manner. The mRNA expression of cholesterol 7-α-hydroxylase 1 (CYP7A1) was decreased in hypercholesterolemic mice and increased by eritadenine and *L. edodes* (5, 10 and 20%) supplementation. In liver tissues, it was observed that lipid accumulation was reduced by treatment with eritadenine and *L. edodes*. In addition, it was revealed that the formation of atherosclerotic plaques due to the HFD was also suppressed by eritadenine and *L. edodes*. The results of the study indicated that the consumption of an HFD may inhibit CYP7A1 expression in the liver by increasing serum T-CHO, LDL and TG levels. *L. edodes* may help regulate lipid metabolism, suggesting that this fungus ameliorates hypercholesterolemia in mice by regulating CYP7A1 expression in the liver.

## Introduction

*Lentinus (L.) edodes* (also know as shiitake) is a popular mushroom known to possess beneficial bioactivities. This mushroom is an important ingredient in Asian cuisine, due to its desirable taste and odor. In addition, the fungus has high levels of vitamins B and D (1). One of the bioactive compounds in *L. edodes* is eritadenine, which has been demonstrated to exert anti-hypercholemic effects in previous studies (2,3). In addition, eritadenine may regulate lipid metabolism by inhibiting S-adenosyl homocysteine hydrolase activity (4–6).

The conversion of cholesterol into bile acids in the liver is important for eliminating cholesterol from the body. Bile acids are essential for the absorption and transport of lipid-soluble vitamins, steroids and xenobiotics (7–9). The main bile acid biosynthetic pathway is initiated by cholesterol 7-α-hydroxylase 1 (CYP7A1), a cytochrome P450 heme enzyme (10). The transcription of CYP7A1 is inhibited by bile acid feedback and stimulated by dietary factors, such as cholesterol (11,12). The expression of CYP7A1 is downregulated by sterol regulatory element-binding proteins (SREBPs) when plasma cholesterol levels are low and upregulated by the nuclear receptor liver X receptor (LXR) when cholesterol levels are high (13).

Dyslipidemia and hypercholesterolemia are among the numerous risk factors associated with diabetes and metabolic diseases, including fatty liver, obesity and atherosclerosis (14–16). Eritadenine (an adenosine analog alkaloid) and lentinacin (a purine alkaloid) have been shown to reduce cholesterol levels in rats by 25% following 7 days of oral administration at doses as low as 0.005% of the feed intake (17). The hypocholesterolemic action of eritadenine appears to be associated with the modification of hepatic phospholipid (PL) metabolism, through the induction of phosphatidylethanolamine N-methyltransferase deficiency (4). Dietary eritadenine also alters the fatty acid and molecular profiles of the liver and plasma by suppressing the conversion of linoleic acid into arachidonic acid and decreasing Δ6-desaturase activity (5). In the present study, the effects of *L. edodes* were investigated in a mouse model of hypercholesterolemia induced by the consumption of a high-fat diet (HFD) during the growth stage of the animals (between 4 and 6 weeks of age). The serum levels of total cholesterol (T-CHO) and triglyceride (TG) were measured. In addition, the expression of CYP7A1 in the liver was evaluated in order to study potential mechanisms underlying the hypocholesterolemic effects of *L. edodes*.

## Materials and methods

### Experimental animals

Imprinting Control Region (ICR) mice (5-week-old) were obtained from Koatech (Pyeongtaek, Republic of Korea). All animals were housed in polycarbonate cages and acclimated in an environmentally controlled room (23±2°C, 50±10% relative humidity, frequent ventilation and a 12-h light/dark cycle) prior to use. The mice (n=60) were divided into six groups (n=10 per group). Hypercholesterolemia was induced in five of the groups by the consumption of an HFD (D12336; Research Diets, Inc., New Brunswick, NJ, USA) for 2 weeks (from 4 to 6 weeks of age).

To assess the ability of *L. edodes* to protect against hypercholesterolemia, the mice were fed the HFD alone as a negative control (NC), HFD with 10 mg/kg eritadenine (Santa Cruz Biotechnology, Inc., Santa Cruz, CA, USA) as a positive control (PC) and HFD supplemented with 5% (LE5; w/w), 10% (LE10; w/w) or 20% (LE20; w/w) *L. edodes* for 4 weeks (from 5 to 9 weeks of age). The remaining group (vehicle) was fed with D12337 pellets (Research Diets, Inc.) as a vehicle control. The compositions of the HFD (D12336) and control diet (D12337) are shown in Table I. Body weight (BW) was measured prior to and following the experimental feeding period. All animal experimental procedures were approved by the Ethics Committee of Chungbuk National University (Cheongju, Republic of Korea).

### Serum and urine collection and biochemical analysis

Blood was collected from the abdominal aorta in each mouse, transferred to serum separator tubes (Microtainer tubes; Becton-Dickinson Co., San Jose, CA, USA) and centrifuged at 400 × g for 15 min. The serum was collected and stored in 200-ml aliquots. T-CHO, high-density lipoprotein (HDL), low-density lipoprotein (LDL) and TG levels in the serum were measured using a Hitachi 7080 auto-analyzer (Hitachi Science System Ltd., Ibaraki, Japan).

### RNA extraction and quantitative polymerase chain reaction (qPCR)

Total RNA was extracted from the mouse liver using TRIzol^®^ reagent (Invitrogen Life Technology, Carlsbad, CA, USA), in accordance with the manufacturer’s instructions. RNA concentrations were measured with a microplate spectrophotometer (Epoch; BioTek Inc., Winooski, VT, USA) at 260 nm. RNA quality was evaluated using electrophoresis in 1% agarose gels. Total RNA (1 mg) was reverse transcribed into first-strand complementary DNA (cDNA) using Moloney murine leukemia virus reverse transcriptase (Invitrogen Life Technology) and random primers (9-mer; Takara Bio, Otsu, Japan). Each cDNA sample (1 ml) was amplified with 10 ml 2X SYBR^®^ Premix Ex Taq™ (Takara Bio) and 10 pmol of each primer. Amplification was performed in a 7300 Real-Time PCR System (Applied Biosystems, Foster City, CA, USA) with the following parameters: denaturation at 95°C for 5 min followed by 40 cycles of denaturation at 95°C for 30 sec, annealing at 60°C for 30 sec and extension at 72°C for 45 sec. The sequences of the oligonucleotide primers used for this study were 5′-TCC ACC TTT GAT GAC ATG GA-3′ (sense) and 5′-TTG GCC AGC ACT CTG TAA TG-3′ (antisense) for CYP7A1 (product size, 171 bp); and 5′-CCA GGG TTT GGA ATT ATT TC-3′ (sense) and 5′-GAA GAT AAA CCC TAA GGC TC-3′ (antisense) for 1A (product size, 297 bp). The relative expression levels of CYP7A1 in each sample (normalized to that of 1A) were determined using RQ software (Applied Biosystems). All qPCR experiments were repeated twice.

### Histological staining and analysis of liver tissues

Liver tissues were recovered, frozen in liquid nitrogen and stored at −80°C until use. Frozen liver tissues were cut with a cryomicrotome (CM3050S; Leica Biosystems, Nussloch, Germany) into 5-mm thick sections, stained with Oil Red O (Sigma-Aldrich, St. Louis, MO, USA) and counterstained with Harris hematoxylin (Sigma-Aldrich). Aortic tissues were embedded in paraffin prior to sectioning (5-μm thick). The sections were deparaffinized in xylene, hydrated in descending grades of ethanol and then stained with Harris hematoxylin and eosin (Sigma-Aldrich). The stained sections were viewed and photographed with a microscope (BX51; Olympus, Tokyo, Japan) equipped with a digital camera (DP71; Olympus). All images were captured at ×200 magnification.

### Data analysis

Data are presented as the mean ± standard error of the mean (SEM) and were analyzed with a one-way analysis of variance (ANOVA) followed by Tukey’s multiple comparison test. Statistical analyses were performed using Prism Graph Pad (version 4.0; GraphPad Software Inc., San Diego, CA, USA). P<0.05 was considered to indicate a statistically significant difference.

## Results

### Effects of L. edodes on BW and daily dietary intake

The differences in BW and daily food intake between mice fed the control diet and those given an HFD for 4 weeks were evaluated. The mice from all six groups had similar BW gains, as shown in Table II. The BWs of the mice in the vehicle, NC and PC groups were all the same at the end of experiment. Groups fed with *L. edodes* (5, 10 and 20%) also had similar BW gains compared with those of the other groups at 9 weeks of age.

### Serum levels of T-CHO, HDL, LDL and TG in the hypercholesterolemic mice

Dietary supplementation with *L. edodes* reduced the serum levels of T-CHO, HDL, LDL and TG, as shown in Table III. At 4 weeks, the mice fed the HFD showed a marked increase in T-CHO levels (to 285.2±23.3 compared with 151.5±17.7 mg/dl in the vehicle group). Dietary supplementation with eritadenine (10 mg/kg) or *L. edodes* (5, 10 and 20%) attenuated the rise in serum T-CHO, LDL and TG levels caused by consumption of an HFD. In the groups fed with *L. edodes* (5, 10 and 20%), the levels of T-CHO, LDL and TG were reduced in a dose-dependent manner compared with those in the NC group.

### Expression of CYP7A1 mRNA in the liver of hypercholesterolemic mice

**Figure 1:**
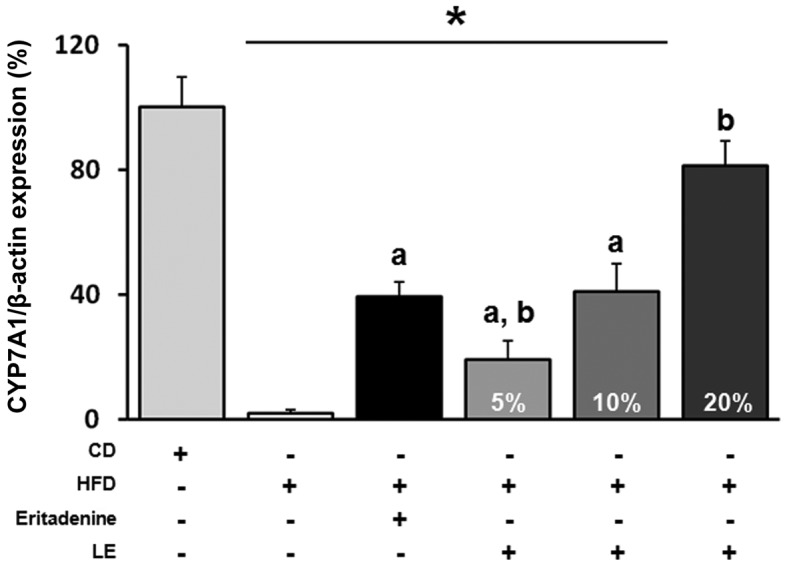
Effects of *Lentinus (L.) edodes* on cholesterol 7-α-hydroxylase 1 (CYP7A1) mRNA expression in hypercholesterolemic mice. Quantitative polymerase chain reaction (qPCR) was performed to monitor the effects of *L. edodes* dietary supplementation on CYP7A1 mRNA expression in the liver of hypercholesterolemic mice. Liver tissues were from six groups of mice fed different diets: i) normal control diet (vehicle; CD); ii) high-fat diet (HFD) alone (negative control; NC); iii) HFD + eritadenine (positive control); iv) HFD + 5% (w/w) *L. edodes* (LE5); v) HFD + 10% *L. edodes* (LE10); and vi) HFD + 20% *L. edodes* (LE20). ^*^P<0.05 compared with the vehicle group; ^a^P<0.05 compared with the NC; ^b^P<0.05 compared with the PC.

The expression of hepatic CYP7A1 was measured in the six groups of mice. As seen in Fig. 1, the level of CYP7A1 mRNA was reduced by HFD administration in the NC group compared with the level in the vehicle group and was significantly increased by eritadenine supplementation in the PC group. Similar to the PC group, hepatic CYP7A1 mRNA expression was increased by *L. edodes* supplementation in a dose-dependent manner.

### Effects of L. edodes on the histology of hypercholesterolemic mouse liver

**Figure 2:**
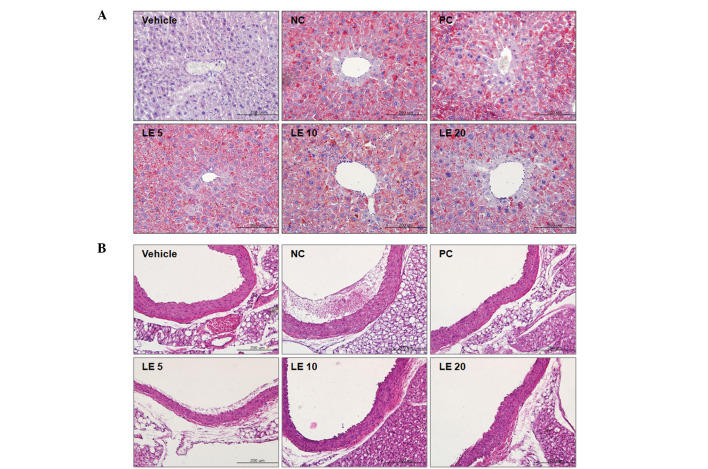
Effects of *Lentinus (L.) edodes* on hypercholesterolimic mouse liver and aorta histopathology. (A) Liver sections (stained with Oil Red O); (B) aorta sections (stained with hematoxylin and eosin). Vehicle, control diet alone; negative control (NC), high-fat diet (HFD) alone; positive control (PC), HFD + eritadenine; LE5, HFD + 5% *L. edodes*; LE10, HFD + 10% *L. edodes*; LE20, HFD + 20% *L. edodes*. Magnification, ×200.

The effect of *L. edodes* on liver tissue morphology in hypercholesterolemic mice is presented in Fig. 2A. Liver tissues were stained with Oil Red O and hematoxylin without eosin. The hepatic cords were arranged in a typical manner and located near the central vein in all groups of mice. Lipid droplets, evidence of fat accumulation in the cytoplasm of hepatocytes, were observed in the livers of hypercholesterolemic mice fed the HFD. The lipid accumulation was reduced by eritadenine or *L. edodes* supplementation.

### Effects of L. edodes on the aortae of hypercholesterolemic mice

The effect of *L. edodes* on the descending aortic tissues of hypercholesterolemic mice is shown in Fig. 2B. Descending aortic tissues were stained with hematoxylin and eosin. Atherosclerotic plaques were observed in mice fed the HFD and the atherosclerotic plaques observed in the PC mice were smaller than those in the mice fed the HFD alone. The atherosclerotic plaques observed in the mice fed with the HFD and *L. edodes* supplementation were smaller and/or less numerous compared with the plaques observed in the NC (and/or PC) mice.

## Discussion

*L. edodes* contains relatively high concentrations of vitamins D, B6, B9 and B12, as well as other beneficial compounds, such as eritadenine and dietary fiber (1). This mushroom has been proposed to be useful for treating hypercholesterolemia, inflammation, hypertension and osteoporosis (18–20). In a previous study, we examined the anti-osteoporotic effects of *L. edodes* and the ability of this fungus to induce the expression of duodenal and renal calcium transport channels in mice with osteoporosis-like symptoms (20). In the present study, we investigated the hypolipidemic effects of *L. edodes* and determined whether this fungus was able to prevent increases in T-CHO, LDL and TG levels in mice fed an HFD. Having fed the mice the experimental diets for 4 weeks, it was observed that dietary *L. edodes* supplementation reduced serum lipid levels, as well as fat accumulation, in the liver and descending aorta of the hypercholesterolemic mice. Eritadenine was demonstrated to have a similar effect. Previously, eritadenine was shown to lower phosphatidylcholine levels in rats (3,21). An analysis of the eritadenine levels in *L. edodes* showed that the concentration of this compound ranged between 3.2 and 6.3 mg/g of dried mushrooms (22).

The stimulation of murine CYP7A1 gene expression by dietary cholesterol has been well studied (23–25). Oxysterols regulate the activity of responsive promoters in this gene by activating nuclear receptors known as LXRs (25). In addition, inactivation of the murine gene encoding the LXRα isoform has been shown to result in resistance to the stimulation of CYP7A1 gene expression by dietary cholesterol (23). CYP7A1 is the key regulatory enzyme of bile acid synthesis, plays a crucial role in cholesterol metabolism and has been implicated in genetic susceptibility to atherosclerosis (26). A CYP7A1 polymorphism has also been demonstrated to increase the progression of atherosclerosis in male patients (27). Furthermore, the development of fatty liver and hypercholesterolemia has been prevented by regulating the expression of CYP7A1 (28).

In the present study, atherosclerotic plaques were observed in the descending aortae of hypercholesterolemic mice. Plaque formation induced by the consumption of an HFD was effectively reduced by *L. edodes* supplementation (Fig. 2B). Hepatic fat accumulation was also observed in the livers of hypercholesterolemic mice. This was inhibited by eritadenine and *L. edodes* supplementation. Furthermore, CYP7A1 mRNA expression was decreased in the hypercholesterolemic mice and upregulated by eritadenine, as well as by *L. edodes* (5, 10 and 20%), in a dose-dependent manner compared with the levels in the NC group.

In conclusion, the present study demonstrated that supplementation with eritadenine and *L. edodes* significantly inhibited the development of hypercholesterolemia induced by an HFD. The reductions in serum lipid levels, hepatic fat accumulation and aortic atherosclerotic plaque formation in mice treated with *L. edodes* illustrated the beneficial effects of this fungus. In addition, dietary supplementation with eritadenine or *L. edodes* increased the level of hepatic CYP7A1 expression in the hypercholesterolemic mice up to that observed in the vehicle control. Based on these results, we propose that the beneficial compounds present in *L. edodes* may be used in the treatment of hypercholesterolemia.

## Figures and Tables

**Table I:** Composition of the purified high-fat (D12337) and control (D12336) diets.

	Diet			
	
	D12336		D12337	
		
Ingredient	grams	kcal	grams	kcal
Casein, 30 Mesh	75	300	75	300
Soy protein	130	520	130	520
DL-Methionine	2	8	2	8
Corn starch	275	1100	522.5	2090
Maltodextrin	150	600	150	600
Sucrose	30	120	30	120
Cellulose, BW200	90	0	90	0
Soybean oil	50	450	50	450
Cocoa butter	75	675	0	0
Coconut oil, 76	35	315	0	0
Mineral mix S10001	35	0	35	0
Calcium carbonate	2.2	0	5.5	0
Sodium chloride	8	0	8	0
Potassium citrate	10	0	10	0
Vitamin mix V10001	10	40	10	40
Choline bitartrate	2	0	2	0
Cholesterol, USP	12.5	0	0.3	0
Sodium cholic acid	5	0	0	0
FD&C Red Dye #40	0.1	0	0	0
FD&C Blue Dye #1	0	0	0.1	0
Total	1000.1	4126	1120.4	4126

**Table II:** Body weights and daily food intake.

Group	Baseline body weight (g)	Body weight 4 weeks later (g)	Daily food intake (g)
Vehicle	36.9±0.5	41.5±1.8	3.77±0.8
NC	36.0±1.3	42.3±0.4	3.24±0.8
PC	35.6±2.1	43.5±0.3	3.83±1.7
LE5	35.8±2.8	42.3±0.3	4.16±1.1
LE10	35.4±2.1	42.1±0.6	4.00±1.2
LE20	35.3±1.5	41.7±0.4	4.12±1.1

LE5, 5% *L. edodes;* LE10, 10% *L. edodes;* LE20, 20% *L. edodes*.

**Table III:** Values of serum chemistry.

	Serum level (mg/dl)					
	
Variable	Vehicle	NC	PC	LE5	LE10	LE20
Total cholesterol	151.5±17.7	285.2±23.3^a^	239.2±15.8^b^	231.5±23.4^b^	182.7±18.0^b^,^c^	147.5±20.2^b^,^c^
HDL-cholesterol	92.8±7.2	92.1±16.4	108.7±13.3	82.8±19.7	97.2±12.0	88.5±15.6
LDL-cholesterol	7.1±3.0	53.8±1.5^a^	45.6±2.0^b^	42.6±7.4^b^	37.8±3.6^b^,^c^	24.5±3.7^b^,^c^
Triglyceride	8.2±1.4	33.5±9.4^a^	23.2±5.0^b^	13±3.0^b^,^c^	9.6±1.1^b^,^c^	7.3±0.7^b^,^c^

a P<0.05 vs. vehicle;

b P<0.05 vs. negative control (NC);

c P<0.05 vs. positive control (PC).

LE5, 5% *L. edodes;* LE10, 10% *L. edodes;* LE20, 20% *L. edodes*; HDL, high-density lipoprotein; LDL, low-density lipoprotein.
